# A larger sized cup accelerates cartilage erosion of acetabulum after bipolar hemiarthroplasty in elderly with femur neck fracture

**DOI:** 10.1097/MD.0000000000029081

**Published:** 2022-04-08

**Authors:** Saumil Ashvin Shah, Jae Young Kim, Hyun-Woo Cho, Won-Yong Shon, Sang-Min Kim

**Affiliations:** a Department of Orthopedic Surgery, Korea University College of Medicine, Guro Hospital, Seoul, Korea; b Department of Orthopedic Surgery, Bumin Hospital, Pusan, Korea.

**Keywords:** acetabular erosion, bipolar hemiarthroplasty, elderly, femur neck fracture

## Abstract

Bipolar hemiarthroplasty (BHA) is one of the common procedures done for the treatment of femur neck fracture. One of the frequently encountered complication with this surgery is erosion of the acetabular cartilage. This study was conducted to investigate acetabular erosion after BHA according to the difference in diameter between femoral head and implanted cup at minimum 10-year follow-up.

We retrospectively reviewed 117 patients (117 hips) undergoing BHA with fracture of neck of the femur. Their mean age was 77.8 years (range, 65–96 years) and male: female ratio was 32:85. Patients were divided into 3 groups; Group A – bipolar cup size > actual head size, Group B – cup size < head size, Group C – cup size = head size. The degree of both superior and medial acetabular cartilage erosion was identified and calculated on postoperative radiographs using line of acetabular margin and Kohler line.

The mean superior and medial acetabular erosion were 1.62 ± 1.6 mm (range, 0–4.4) and 4.15 ± 2.7 mm (range, 0–8.2) in Group A, 1.30 ± 1.3 mm (range, 0–3.8) and 4.11 ± 2.7 mm (range, 0–7.8) in Group B, and 0.90 ± 1.1 mm (range, 0–2.6) and 3.16 ± 2.9 mm (range, 0–7.9) in Group C (*P* = .039 and *P* = .187, respectively). The superior acetabular erosion showed significant difference between the 3 groups. During mean follow-up period of 12.3 years, 5 patients (5/117, 4.3%) underwent conversion to total hip arthroplasty due to superior acetabular erosion. All of 3 patient underwent BHA with a larger bipolar cup than the actual femoral head.

A lager sized cup accelerated superior cartilage erosion of acetabulum after BHA. An optimal cup size should be considered when undergoing BHA in elderly patients.

## 1 Introduction

Fracture of the neck of femur is the most common fracture pattern in elderly population around the world.^[1]^ Ha et al^[2]^ reported 4.3% increase in incidence of hip fractures per annum nationally. It is also one of the major causes of morbidity and mortality in elderly population. The treatment of choice for displaced fracture neck of femur is surgery. Despite general consensus to the favorable outcomes of arthroplasty over fixation, the better option between the bipolar hemiarthroplasty (BHA) and total hip arthroplasty (THA). The BHA may be satisfactory for low-demand patients with lots of comorbidities, while THA may lead to better functional outcomes for more active, independent patients. However, most patients with fracture neck femur are the elderly population who have multiple co-morbidities. In such patients, the BHA is considered a reasonable option to regain good function.^[3]^ It is preferred as it requires less operative time, less blood loss, technically easier procedure than THA.^[4]^ It also has a relatively lesser dislocation rate compared to THA.^[5,6]^

However, there is a risk of cotyloiditis after BHA which is defined as progressive erosion of acetabular cartilage and bone erosion secondary to friction between the hard head of the prosthesis and the soft articular surface of the acetabulum.^[7,8]^ Postoperative groin pain is a potential complication and can lead to serious morbidity due to increased lifespan. It leads to severe limitation of autonomy and may require revision. It has a multifactorial causation. However, there is limited literature available specifically evaluating the cause of acetabular erosion.

Aim of this study was to investigate and compare clinical and radiological outcomes in patients treated with BHA with a small, large and same size cup than the size of the femoral head. In particular evaluate the incidence of acetabular erosion in patients with a larger cup.

## 2 Patients and methods

Data from our hip replacement registry, to which single orthopaedic surgeon report was utilized after obtaining approval from Korea University Institutional Review Boards. All methods were carried out in accordance with relevant guidelines and regulations. Written informed consent was obtained from all subjects.

**Figure 1:**
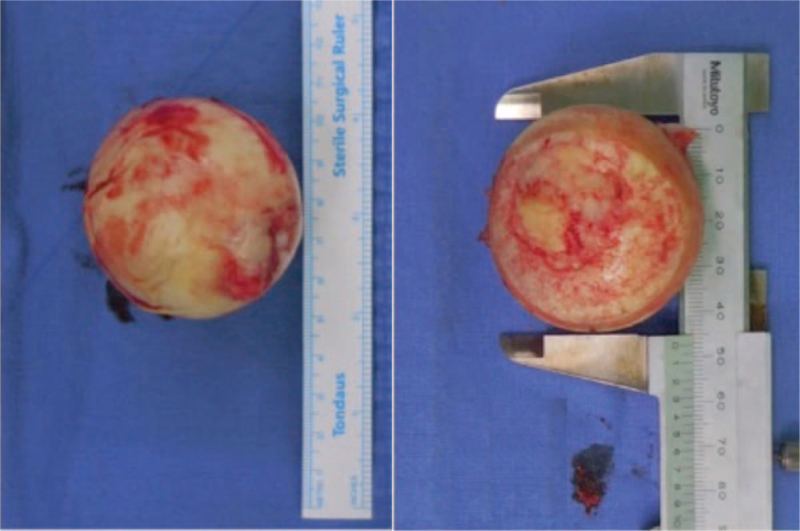
Measurement of size of femoral head (A) excised femoral head. (B) Measurement of Antero-posterior and medio-lateral diameter using a caliper at the level of maximum diameter.

**Figure 2:**
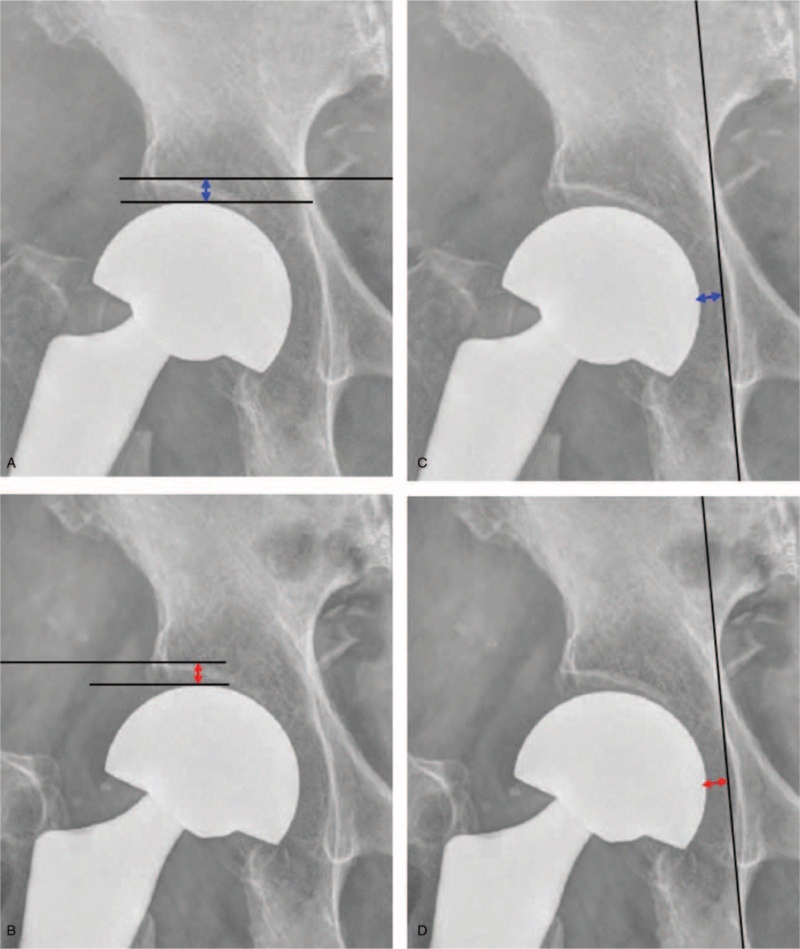
Measuring the amount of acetabular erosion. (a) Immediate postoperative radiograph measuring the distance from acetabular line to the level of prosthesis (A). (b) Follow-up radiograph measuring the same distance (B). (c) Immediate postoperative radiograph measuring the distance from Kohler line to prosthesis (C). (d) Follow-up radiograph measuring the same distance. Superior acetabular erosion calculated as (A and B) in mm. Medial acetabular erosion calculated as (C and D) in mm.

For each patients, following data was recorded: demographic data, co-morbidities, American Society of Anesthesiologists^[9]^ score, time of fracture to surgery, duration of surgery, type of implant used, and the size of femoral head. All patients were operated for cementless BHA. Surgical approach was posterolateral approach in lateral position. The femoral head size of femur was calculated using caliper (Fig. 1) and accordingly the cup size was used as a trial. Based on the stability, the size of the cup was increased or decreased by 1 mm. Postoperatively, patients were mobilized with walker on day 1 and physiotherapy was started. Regular follow-ups were undertaken at 6 weeks, 3, 6, and 12 months and thereafter, annually. At each follow-up, Harris Hip score (HHS) and radiographs of both hip AP view as well as translateral hip view were taken. The radiographs were compared with day 0 postoperative radiographs. The superior acetabular erosion was measured by measuring the migration of the femoral head using the acetabular line which is a line joining the outer tip of both acetabuli. The medial acetabular erosion was calculated by using the Kohler line (Fig. 2).^[10]^ The erosion was recorded in millimeters and grouped according to the size of the prosthesis compared with the size of the femoral head. The patients who had a bipolar cup size more than the size of the femoral head were grouped in Group A. Patients with a cup size smaller than the size of the femoral head were grouped as Group B and the patients who had a cup size equal to the size of the femoral head were grouped as Group C. The acetabular erosion and functional outcomes including HHS^[11]^ and pain visual analog score were compared among each group.

The Statistical analysis using SPSS version 23.0 was performed. Descriptive statistics including means and minimum and maximum were computed to summarize the distribution of continuous variables, while frequencies and percentages were computed for categorical variables. The independent *t* test was used to compare continuous variables, and the Mann–Whitney test or Fisher exact test was used to compare categorical variables. Statistical significance was defined at *P* ≤ .05.

## 3 Results

Our institution's database containing prospectively collected demographics, surgical data, and patient outcomes identified 176 patients undergoing cementless BHA with a diagnosis of fracture of neck of the femur between 2004 and 2010. The inclusion criteria of the study were age more than 60 years, Garden^[12]^ type fracture grades III/IV, ambulation of at least 500 m prior to injury. The patients who were unable to ambulate, pathological fracture, arthritic changes in the hip were excluded from the study. Forty-five patients were lost to follow-up or dead. Fourteen patients underwent revision surgery due to loosening, fracture, dislocation, or infection. The final study cohort comprised of 117 patients (117 hips). Postoperative mean follow-up period was 12.3 years (range, 10.0–16.8 years).

Patient demographics are shown in Table 1. All patients underwent cementless BHA. The degree of superior acetabular erosion was higher for Group A (the size of bipolar cup > the actual size of head), compared to other groups (the size of bipolar cup ≤ the actual size of head) (Table 2, *P* = .187). The degree of medical acetabular erosion was also higher for Group A, but the difference was not significant (*P* = .187).

**Table 1:** Baseline patients characteristics.

Age (yr) mean	77.75 (55–96)
Gender	
Male	32 (58–96)
Female	85 (55–84)
Body mass index (mean)	22.06 (13.52–31.25)
ASA grade (1/2/3/4) 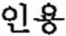	16/59/39/3
Cup size	
Group A^∗^/Group B^†^/Group C^‡^	41/27/49

ASA = American Society of Anesthesiologists.

∗ Size of bipolar cup > actual size of head.

† Size of bipolar cup < actual size of head.

‡ Size of bipolar cup = actual size of head.

**Table 2:** Results in group of patients with degree of erosion.

	Group A (N = 41)	Group B (N = 27)	Group C (N = 49)
Superior acetabular erosion^∗^			
Mean	1.62	1.30	0.90
Std deviation	1.6	1.3	1.1
Range	0–4.4	0–3.8	0–2.6
Medial acetabular erosion^†^			
Mean	4.15	4.11	3.16
Std deviation	2.7	2.7	2.9
Range	0–8.2	0–7.8	0–7.9

Std = standard deviation.

∗ *P* value = .039.

† *P* value = .187.

The average hip score was a 77.21 (48–91). In the study population, the overall HHS was 77.2 ± 12.7 (range, 48–91). The mean HHS was 78.17 for Group A, 75.53 for Group B, 77.12 for Group A, respectively (*P* = .650) at final follow-up. The mean pain visual analog score was 34.43 for Group A, 31.85 for Group B, 35.06 for Group C, respectively (*P* = .330).

In our series, 38% patients had no difficulty in squatting position and were able to do successfully, 40% patient had some difficulty but were able to do it. Remaining 22% patients were not able to squat. Forty percent of the patients were able to sit cross-legged without any difficulty, 45% had some difficulty while sitting cross-legged and 15% patients were not able. However, there are no cases of dislocations seen during the study.

**Figure 3:**
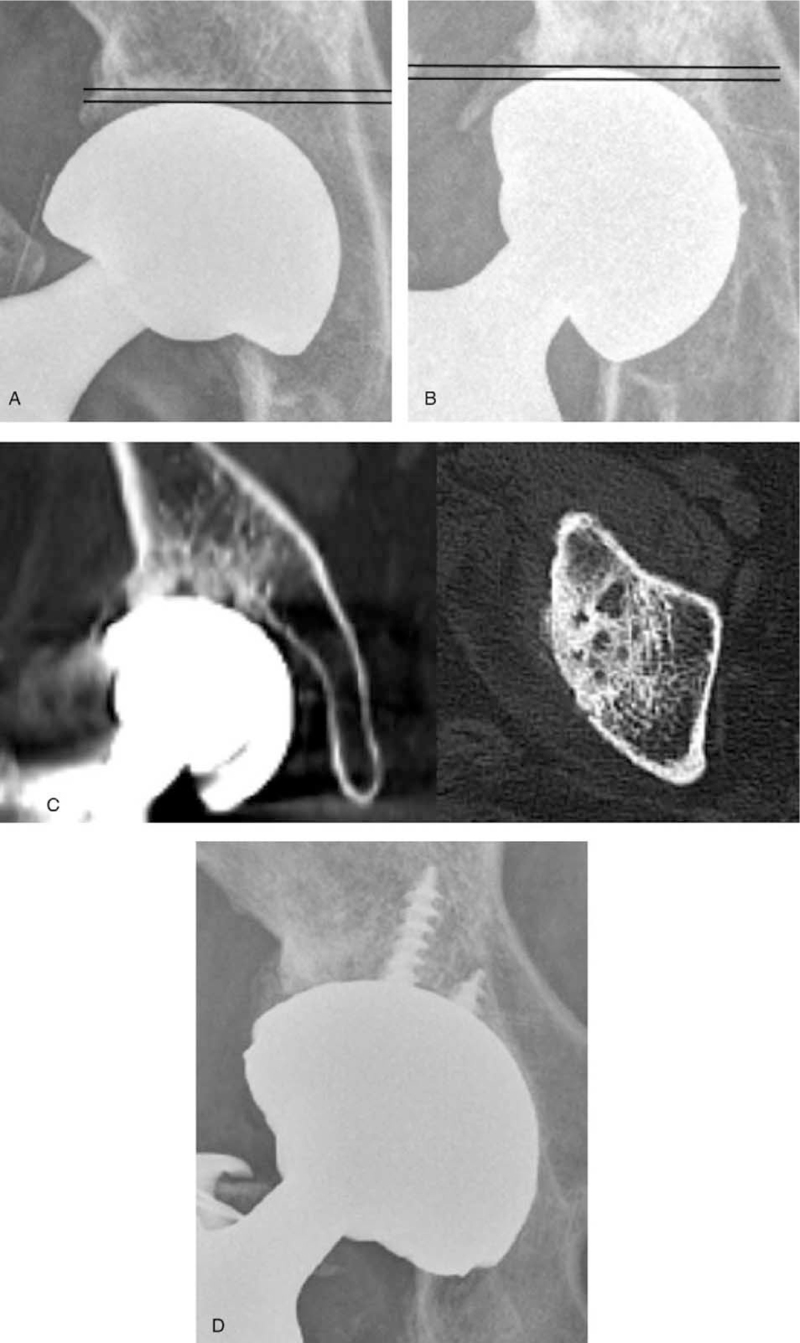
(A) A 80-year-old woman underwent bipolar hemiarthroplasty with femur neck fracture. (B) At 3 yr postoperatively, the bipolar cup was migrated superior up to the 3.8 mm. (C) Subchondral erosion with sclerotic change and cyst formation is seen on computed tomography. (D) She underwent conversion to total hip arthroplasty.

During mean follow-up period of 12.3 years, 5 patients (5/117, 4.3%) underwent conversion to THA due to superior acetabular erosion (Fig. 3). All of 3 patients were in Group A (the size of femoral head prosthesis > the actual size of head). They complained severe inguinal pain on walking and standing from sitting position.

## 4 Discussion

In the elderly population with fracture of neck of the femur, BHA or THA are the preferred mode of surgery of most orthopedic surgeons. The postoperative autonomy is retained faster in the arthroplasty group, compared to osteosynthesis group.^[13]^ However, there are increased complications following arthroplasty of the hip joint.^[14,15]^ BHA is generally considered for patients who are having a poor autonomy preinjury. In this study, we have compared the size of the femoral head to the size of the prosthesis and measured the amount of acetabular cartilage erosion and functional outcomes of each group at long-term follow-up.

From this study, we found a significant increase in the amount of superior acetabular erosion following BHA when the size of the prosthesis was larger than the size of actual femoral head (*P*-value, .039). The medical acetabular erosion also increased when the size of the prosthesis was larger than the size of the femoral head, although the trend was not statistically significant. Our data from long-term follow-up demonstrated that the amount of both superior as well as medial acetabular erosion was less, when the bipolar cup size was well matched with the actual femoral head.

Animal studies have proven advanced degenerative changes after BHA. At 24 weeks, most of the acetabular articular cartilage is lost.^[16]^ Moon et al^[7]^ estimated mean linear degeneration of acetabular cartilage was measured to be 0.23 ± 0.0107 mm/yr. The amount of degeneration is directly proportional to the actual duration of articulation of the prosthesis to the acetabular cartilage.^[17]^ The rate of acetabular erosion is considered to be multifactorial such as type of material used for the prosthesis, sliding distance, activity level.^[7,18]^ Squires and Bannister^[17]^ had seen increased amounts of erosion of the acetabulum when the prosthetic heads were available in sizes increments of 2 mm leading to increased mismatch of the femoral head and the prosthesis. In a finite element study, a larger clearance in BHA is proven to be more harmful to the acetabular cartilage.^[19]^ They suggested that prosthesis heads with smaller increments in diameter should be manufactured for surgeons to reduce contact stress in BHA surgeries. Thus, we believe the most important factor for acetabular erosion is the size of the prosthesis.

There have been numerous reports suggesting bipolar device ceases to function normally and acts as a unipolar device within 3 to 12 months of surgery.^[20,21]^ Once the intraprosthetic motion is decreased and the joint becomes stiff, there is movement occurring at the level of the head of prosthesis and the acetabular cartilage. It leads to increased motion around the joint leading to increased level of erosion. Such erosion leads to proximal or medial migration of prosthesis and causing increased disability and the need for revision surgery.

Thirty percent of the patients were able to squat after BHA according to a study by Kaur et al.^[22]^ In our study 38% patients had no difficulty while squatting and 40% of the patients were able to sit cross-legged without any difficulty. There were no reported cases of dislocation in our study during follow-up.

This study has some limitations. First, there was a limited number of patients. It is not easy to follow the study subjects with long-term. The high rate of loss of follow-up from the study is inevitable when their advanced age and poor functional status is considered. Nevertheless, on investigation of the studies available regarding acetabular erosion after BHA, the current data results from one of the longest follow-up studies. Second, the retrospective nature of this study has inherent risk of observer bias, including the potential for missing data and inability to control confounding variables. Lastly, metabolic factors like bone quality measurements and bone mineral density was not taken in the study and hence they were not evaluated in the treatment protocol.

In conclusion, 117 patients (117 hips) undergoing BHA were investigated with long-term follow-up. Our data showed that there is accelerated acetabular erosion when the size of the prosthesis is more than the size of the femoral head. During mean follow-up period of 12.3 years, 3 patients (5/117, 4.6%) underwent conversion to THA due to superior acetabular erosion. All of 3 patient underwent BHA with a larger bipolar cup than the actual femoral head. An optimal cup size should be considered when undergoing BHA in elderly patients.

## Author contributions

All authors reviewed the manuscript.

Shah, Sang Min Kim, Cho, & Jae Young Kim wrote the main manuscript text and Shon prepared all figures.

**Writing – review & editing:** Saumil Ashvin Shah, Won Yong Shon, Sang-Min Kim.

**Writing – original draft:** Jae Young Kim, Hyun Woo Cho.
